# Supine arm cycling during the post-flap recovery period for persons with spinal cord injuries: The multi-purpose arm cycle ergometer (M-PACE) safety and pilot testing

**DOI:** 10.1080/10790268.2021.1975082

**Published:** 2021-11-02

**Authors:** Christine M. Olney, John E. Ferguson, Greg Voss, Eric Nickel, Stuart Fairhurst, Alexandra S. Bornstein, Sara Kemmer, Crystal Stien, Kristin Scheel, Charlotte Brenteson, Ann Goding, Mary Murphy Kruse, Byron Eddy, Gary Goldish, Andrew H. Hansen

**Affiliations:** 1 Minneapolis Veterans Affairs Health Care System, Minneapolis, Minnesota, USA; 2 Department of Rehabilitation Medicine, University of Minnesota, Minneapolis, Minnesota, USA; 3 Division of Rehabilitation Science, University of Minnesota, Minneapolis, Minnesota, USA; 4 Department of Biomedical Engineering, University of Minnesota, Minneapolis, Minnesota, USA

**Keywords:** Bedrest, Deconditioning, Ergometry, Exercise, Flap surgery, Pressure injuries, Rehabilitation

## Abstract

**Objective:**

To describe how using a supine arm cycle ergometer can safely reduce deconditioning experienced by patients with spinal cord injury or disorder (SCI/D) during their four to six weeks of complete bed rest after surgery to close a stage 4 pressure injury.

**Design:**

This pilot project used a newly designed arm cycle ergometer (known as the M-PACE) that extends over the bed, allowing a patient to lie completely supine while exercising.

**Setting:**

The M-PACE was designed and built at the Minneapolis Veterans Affairs Health Care System (MVAHCS) and pilot tested at the MVAHCS SCI/D Center.

**Participants:**

Patients with SCI/D, recovering from flap surgery and deemed appropriate to use the arm cycle ergometer were enrolled in the pilot study (*n* = 47).

**Outcome Measures:**

A pre–post six-minute arm test (6MAT), a proxy for conditioning, was conducted on a subset (*n* = 15) of participants before and after the supine cycling exercise training program. Participants’ rating of perceived exertion (RPE) scores were collected at cessation of each 6MAT. Participants gave feedback on their perception of using the M-PACE.

**Results/Conclusions:**

The 6MAT RPE was significantly reduced after training with the M-PACE while on bed rest (*P* = 0.003). Also, significantly more rotations were performed after completing the training program (*P* = 0.02). Further, study participants who accessed the M-PACE found using it helped offset the tedium of laying supine during flap surgery recovery. The differences in the 6MAT pre- to post measures indicate the M-PACE should be further studied for offsetting the normal deconditioning that occurs with extended bedrest.

## Introduction

Surgical repair of a deep stage 4 pressure injury is known as flap surgery. This surgery provides a full thickness tissue covering that can be nearly as resilient to reinjury as the original tissue. A period of immobilization is required after flap surgery to allow the surgical wound to mature enough to tolerate any strain associated with movement. For people with spinal cord injury and disorders (SCI/D), recovery from flap surgery is especially challenging because most stage 4 pressure injuries involve their sitting surfaces (i.e. ischial, sacral, trochanter, and coccygeal areas). Typically, this means a prolonged bedrest, including lying supine, before initiation of daily activities. Prolonged bedrest is known to be highly associated with adverse health outcomes, including deconditioning.^1–4^ This conflict between needed prolonged bedrest for wound healing versus potential adverse health outcomes, drove the development of an exercise device that can be safely used by persons with SCI/D while recovering from flap surgery.

Surgeons and supporting care teams have differing opinions regarding the minimum time needed for a flap wound to tolerate resumption of sitting. In practice, Veterans Affairs SCI/D Centers’ directors within the Veterans Health Administration Spinal Cord Injury System of Care reported requiring 4–6 weeks of strict bed rest following flap surgery.^5^ Given that aerobic capacity decreases approximately 0.85% per day during the first 30 days of bedrest,^6^ and, further, muscle strength decreases as much as 35–50% with 5 weeks of bed rest,^7^ persons with SCI/D recovering from flap surgery are at risk of losing significant upper extremity function essential for self-cares such as transfers, weight shifts for pressure relief and wheelchair mobility. These self-care activities are crucial to their independent living and safety, thus it is not uncommon for people with SCI/D who undergo flap surgeries, to require prolonged hospital stays to complete upper body reconditioning and rehabilitation prior to discharge.

Halar and Bell^7^ stated “the only proven way to prevent and treat disuse atrophy is with exercise”, compelling our health care team to discover safe, effective methods to prevent deconditioning in persons with SCI/D while recovering from flap surgery. Further, we know from McLean and Skinner that the training effect achieved by a supine exercise in people with SCI/D does generalize to the seated posture, suggesting that persons exercising during bedrest in the supine posture will be able to translate the benefits to activities of daily living in a wheelchair.^8^

Once the surgical wound is healed enough to tolerate sitting, post-flap patients start using an arm cycle ergometer in the gym to help regain that which was lost during their recovery period. The Minneapolis Veterans Affairs Health Care System (MVAHCS) SCI/D care team has determined that earlier access to arm ergometry during the post-flap surgery recovery phase could possibly offset deconditioning and lead to an earlier hospital discharge and return to independence.

Options for supine exercise for this patient population while on bedrest are limited. Our therapists at the MVAHCS SCI/D Center have found exercises such as bedside weights and Theraband^®^ have limited success in preventing deconditioning in patients with SCI/D following flap surgery. The exercise provided by arm cycling ergometers is effective, but these devices are not suitable for use when supine in a hospital bed and are therefore limited to use in the gym once patients are able to tolerate sitting. One arm cycle ergometer, called the MOTOmed^®^, does go over the bed but was developed to provide motor or FES assisted arm movements for patients who are too weak or deconditioned to move on their own; however these features are not required in many post-flap recovery patients.

**Figure 1:**
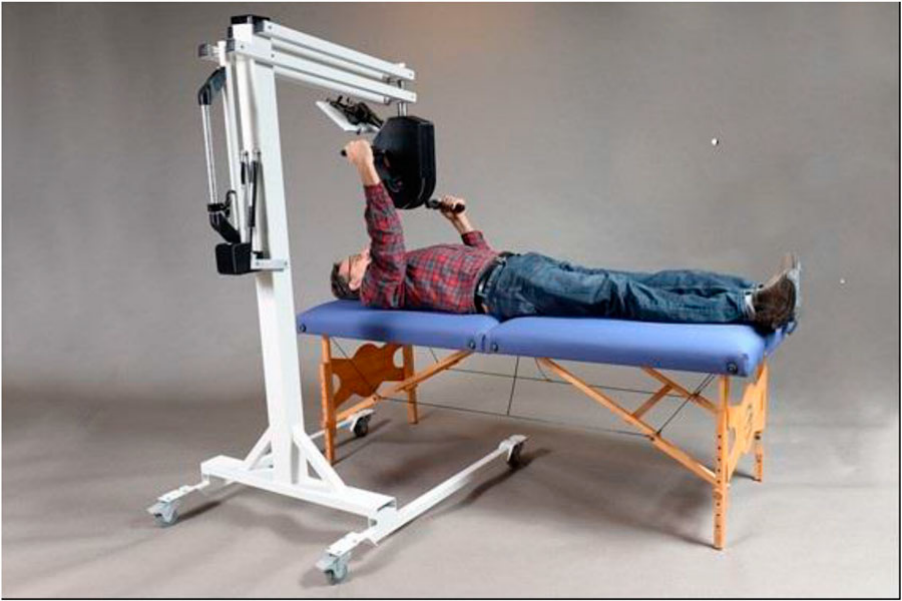
Final design of the supine arm cycle ergometer. The default safety feature moves the overhead mechanism away from the patient.

To address the need for exercise in the supine position, the Minneapolis Adaptive Design and Engineering (MADE) team and other stakeholders developed an arm cycle ergometer, now known as the Multi-Purpose Arm Cycle Ergometer (M-PACE) (Fig. 1), which uses an eddy current brake resistance mechanism allowing it to be safely used while lying supine in a bed, sitting upright in a wheelchair or from a standing position. This paper briefly describes Veterans’ and clinicians’ feedback during the two phases of the development project, the safety issues considered and the pilot pre/post endurance testing within our post-flap recovery program at the MVAHCS SCI/D Center.

## Methods

### Safety testing: Phase I

Approval for conducting research with the M-PACE was obtained from the MVAHCS Institution Review Board. Phase I testing was primarily about safety testing and conducted with Veterans with SCI/D, not recovering from surgery, in the outpatient setting. Measures included peak pressure index and fluctuations in peak pressure index using interface pressure mapping of their pelvic area while arm cycling in the seated position and while lying on the bed surface; rating of perceived exertion (RPE)^9^ while cycling; and qualitative feedback on the device features and performance during supine arm cycling at low and high speeds and low and high resistances.

### Pilot testing: Phase II

Enrollment into Phase II included rigorous screening of each potential participant by the study physician, the wound surgeon and/or the wound nurse, and the study physical therapist or occupational therapist, to ensure the patient was appropriate for the study. Inclusion criteria allowed any Veteran undergoing flap surgery to be considered for enrollment, with the caveat of the care team agreement, and the ability of the patient to lay supine after surgery. Clinical assessment included evaluating co-morbidities of each potential participant, the extent to which those co-morbidities might impact the outcome of the flap surgery, and whether the clinicians believed these co-morbidities may exclude exercise using the M-PACE.

The post-flap protocol at the MVAHCS SCI/D Center includes four to six weeks of complete bedrest with the head of bed flat or slightly raised to 10 degrees. The Phase II protocol was conducted during this bedrest period, and length of each session using the M-PACE varied based on the participant’s recovery, which was dependent on type and extent of flap surgery and comorbidities (e.g. heart disease frailty, osteomyelitis, infection, etc.). Typically, the care team would initiate M-PACE use at post-op day seven for ischial flap surgery or on post-op day 14 for sacral flap surgery. This varied start time was a recommendation given by the plastics surgeon and wound specialist and based on concern that the sacral flap surgical wound would have more direct pressure and weight throughout while lying in the full supine position versus an ischial area flap surgical wound which often wraps around the outside of the thigh, and would be less vulnerable to shear and pressure concerns. The care team was the gateway to M-PACE use, always ensuring wound safety. Surgical wounds and all drain outputs were examined prior to initiation of each exercise encounter and after each encounter to ensure there was no impact on the wound. Therapy sessions were anywhere from 15 to 45 minutes usually five days a week, but sometimes less, depending upon factors such as the patient’s interest to be engaged for more or less exercise time, their physical abilities and activity tolerance the therapist’s schedule, or device availability. Our protocol allowed participants to use the M-PACE prior to surgery if they were on prolonged bedrest. Further, though not widely advertised, our protocol allowed for patients other than those with SCI/D to be enrolled to use the M-PACE if for some reason they were unable to access a therapy space.

Outcome data included qualitative feedback from the Veterans and the clinicians implementing the device use. The engineering team made iterative changes over the study period (approximately five years) based on these qualitative data. Further, an efficacy measurement was added in the third year of the study, the six-minute arm test (6MAT)^8^ to begin to understand effects of using the M-PACE during the post-flap recovery bedrest period. A subset of participants, while on bedrest, completed a 6MAT (pre) prior to initiation of M-PACE use and a second 6MAT (post) just prior to the participants’ ability to attend the therapy gym (i.e. at the end of sitting restrictions). With the assistance of a built-in metronome, each participant was encouraged to cycle at 60 rotations per minute, thus the expected number of rotations at the end of the 6MAT was 360 rotations. Each participant practiced the 6MAT the previous day, to ensure they would be able to maintain that speed at a determined level of resistance for the duration of the test. RPE scores were collected at the end of each 6MAT, which were used to represent the conditioning outcome.^10^

### Analysis

Ratings of perceived exertion at the end of the 6MAT and number of rotations during the 6MAT were compared between pre- and post-exercise program conditions using paired *t*-tests. Qualitative data (interviews and user feedback) were routinely logged into the database. Study personnel then briefed engineers regularly regarding needed changes to the M-PACE design and function.

## Results

### Safety testing: Phase I

The safety testing with outpatient Veterans with SCI/D (*n* = 4) focused on determining speed and resistance levels for supine arm ergometry in an effort to provide exercise without causing harm to the post-flap surgery wounds in Phase II. Interface pressure mapping was conducted and demonstrated reduced peak pressures under the ischial tuberosities during supine cycling compared to seated cycling.^11^ In addition, high pressure fluctuations under the pelvis were found when subjects cycled at high speeds, but low fluctuations in pressure when increasing the resistance to cycling.^11^ Phase I participants’ qualitative feedback was consistently positive for the device’s ability to provide access to exercise in the supine posture and all stated that they would “definitely use” the device if they were placed on bedrest.

### Pilot testing: Phase II

Demographics for Phase II of all enrolled participants (*n* = 47) and the subset of participants who were included in the pilot testing (*n* = 15) are found in Table 1. During the study timeframe (July 1, 2014 through December 1, 2019), the MVAHCS SCI/D Center performed 70 flap surgeries, of which 67% were considered appropriate study participants and enrolled prior to surgery to use the M-PACE post-flap surgery. The major reasons the care team did not endorse use of the M-PACE post-flap surgery for 33% of the patients were: poor skin integrity, unmitigable pain in arm(s) or shoulder(s), not good enough arm function, inappropriate behavior, or the patient was not interested in participating.

**Table 1:** Demographics for Phase II: All and Subset.

	Phase II (All)*	Phase II (Subset)^#,***^
Non-SCI/D	1^+^	0
SCI/D		
Tetraplegia	15	3
Paraplegia	31	12
Age (ave. yrs.)	62**	64
Cause of injury		
MVA	27	10
Fall	6	3
Other	13	2
Type of wound^§^		
Sacral	9	3
Coccyx	1	1
Ischial	24	9
Trochanter	7	0
Other	3	3

*Four participants included had two different flap surgeries during 5-year study period, thus considered separate events.

**Ages of the four repeats are included in this average.

^#^ Subset of *n* = 15 are part of the full set *n* = 47.

***There were no repeated surgeries in this subset group.

^+^ This participant was unable to access regular gym due to morbid obesity, thus used M-PACE during post knee surgery rehabilitation.

^§^ Several participants had two types of wounds repaired with their flap surgery.

During early Phase II testing, the MADE team continued to improve the M-PACE design and function. Both post-flap surgery patients and the study clinicians continually offered comments and feedback to the engineering team. Examples of suggested changes included requests to improve the stability of the M-PACE during cycling and improve the ability to maneuver the viewing screen for therapist and patient. Figure 1 shows the final M-PACE iteration, with only minor changes occurring thereafter. The study team began using this iteration about halfway through recruitment period, with the 22nd participant being the first to use this final iteration.

Phase II (inpatient) enrolled 47 participants of which 35 used the M-PACE during post-flap surgery recovery. Eight participants did not have flap surgery after being consented due to assorted reasons (e.g. wound was able to heal without surgery, participant condition changed) but four of those eight participants used the M-PACE during treatment and management of their wounds. One patient, without a SCI/D, was enrolled to use the M-PACE because morbid obesity prevented access to any other exercise device while on bedrest. There were 11 (*n* = 11) withdrawals over the 5 and one-half years of conducting the pilot study. Of those withdrawals, eight (*n* = 8) were withdrawals by the study team and three were participants withdrawing themselves. See Table 2 for reasons for withdrawals.

**Table 2:** Reasons for withdrawals (*n* = 11).

Withdrawal ST or PPT*	*N*	Rationale
ST	4	Did not have flap surgery and did not use M-PACE
ST	1	Developed wound infection
ST	2	Fragile surgery
ST	1	Post-flap cardiac arrest; never used M-PACE
PPT	1	Decided to not use for unknown reason
PPT	1	Did not like shoulder and abdominal soreness after use
PPT	1	Was worried Exercise might harm the wound

*Withdrawal by Study Team (ST) or Participant (PPT).

Feedback from participants was predominately positive and included statements such as: “it was actually the one and only thing I was able to look forward to while going through the dreadful process of getting a flap!”; “I like using the bike, it’s better than laying here and feeling like I’m turning to mush.”; “I didn’t realize my arms were so weak.”; “I want to keep at it.”; “I like the bike  …  it’s nice to have something to do.”; “I always look forward to using it”; “I still am not nearly where my endurance and strength should be … I really enjoyed this bike though.” One participant disagreed with others, stating that using the M-PACE was “boring”.

**Figure 2:**
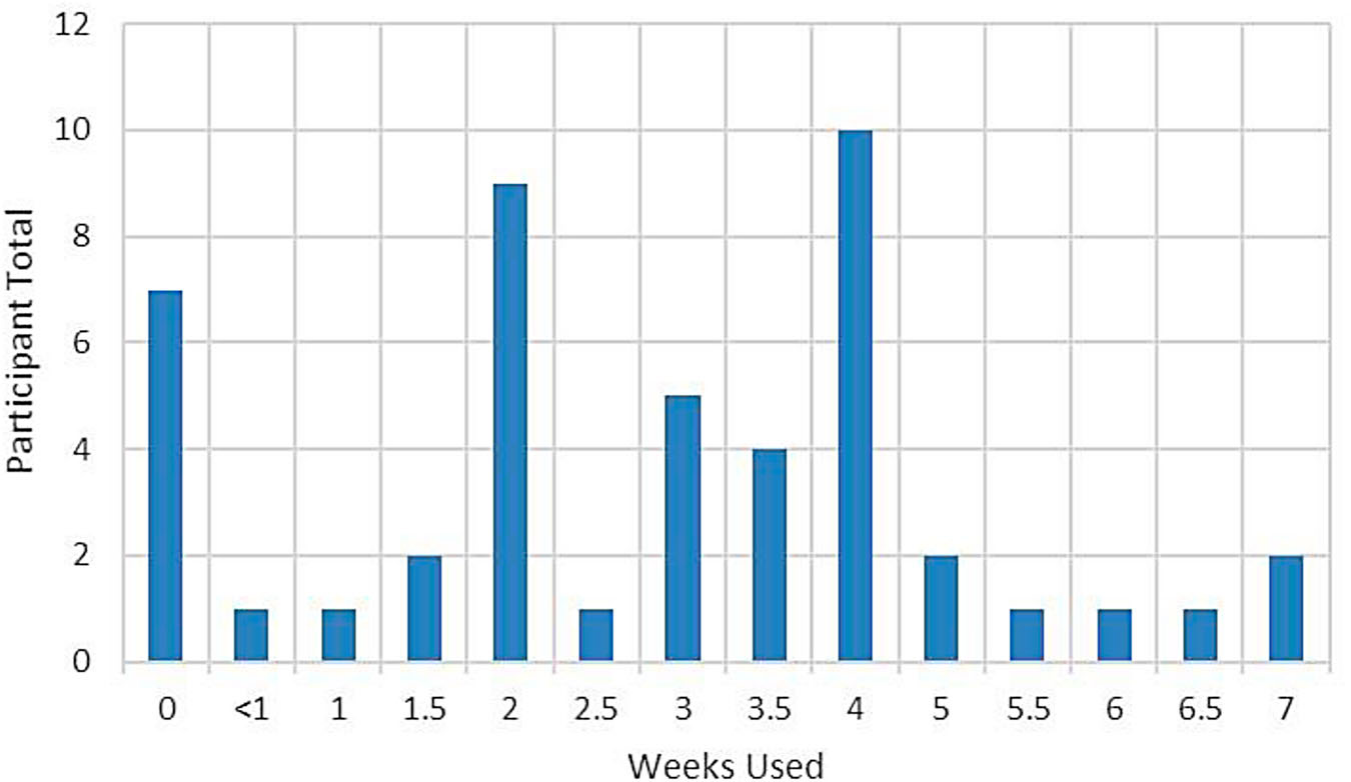
Usage of the M-PACE varied across the participants, depending on their type of flap surgery, prescribed time on bedrest, or other mitigating circumstances. This figure depicts the number of weeks the M-PACE was used by 40 of the 47 participants (39 participants with SCI/D, one non-SCI/D participant).

Usage and frequency varied across participants. To summarize, of the 47 enrolled participants, 40 used the M-PACE at least once (39 participants with SCI/D who were pre- or post-surgery or both, plus the one non-SCI/D participant) and some used the M-PACE up to seven weeks. Figure 2 depicts the M-PACE usage of the 40 participants, with an average of 3.4 weeks usage.

**Figure 3:**
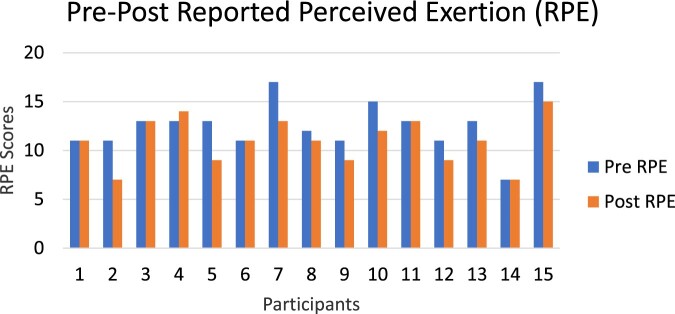
Pre and post reported perceived exertion (RPE) scores at the end of the 6MAT of participants who used the M-PACE after flap surgery (*P* = 0.003). A lower score suggests less exertion after the 6MAT. Of the 15 cases, 14 maintained their conditioning (RPE stayed the same or reduced). Nine of those 14 improved their conditioning as reported per RPE during the post-surgical phase.

**Figure 4:**
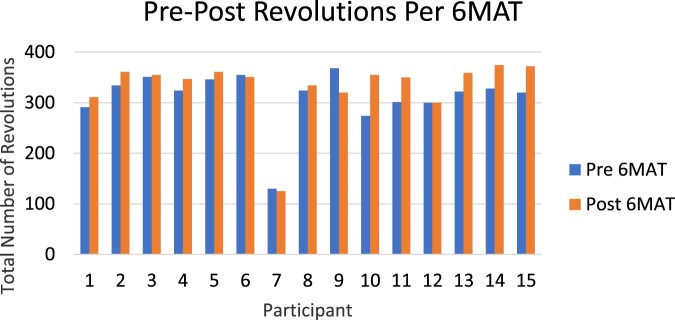
Pre and post revolutions using the M-PACE during the 6MAT. The increase in the number of revolutions pre to post was significant (*P* = 0.02).

The subset of Veterans (*n* = 15) who participated in the 6MAT pilot test used the bike an average of 3 weeks (range 1–5 weeks). Figure 3 depicts the pre- and post-6MAT RPE scores, with significantly less perceived exertion after the exercise program (*t*(14) = 3.53, *P* = 0.003, *d* = 1.5, 95% CI[0.06, 2.47]). Figure 4 depicts the average rotations per 6MAT for each of the participants, showing significantly more rotations during the 6MAT performed after the exercise program (*t*(14) = 2.58, *P* = 0.02, *d* = 20.5, 95% CI [3.45, 37.48]). Of the 15 participants who completed pre–post-6MAT, 12 maintained rotational count or increased the number of rotations. Participants 6, 7 and 9 were not able to maintain their original number of rotations during the post-6MAT. Exercise using the M-PACE was discontinued once the Veteran was no longer bedbound.

Participants did not experience any serious adverse events using the supine arm cycle ergometer, although we did have several adverse events to include the tablet falling on a participant due to a design flaw (which was immediately remedied), one participant possibly experiencing autonomic dysreflexia due to misfit hand grip on the ergometer handles (which was immediately addressed and resolved), and several participants found the exercise caused their shoulders to ache after using the M-PACE.

## Discussion

Historically, clinicians and patients have been frustrated with the lack of access to equipment that would offset patient deconditioning during an extended bedrest plan of care. There is convincing evidence that prolonged bedrest, such as the four to six weeks experienced by persons with SCI/D recovering from flap surgery, can and does negatively influence muscle mass and impacts the ability needed for returning to normal activities of daily living (e.g. doing a safe transfer or conducting full pressure reliefs). Throughout the development of the M-PACE, clinicians and patients have been very eager to use the supine arm cycle ergometer to help offset the prolonged bedrest deconditioning trajectory.

Safety and the post-flap surgical wound protection were at the forefront of every team member’s decisions and actions throughout the design, development, and testing of the M-PACE. Our early work^11^ suggested that using higher resistance and lower rotational speeds (60 cycles per minute or less) demonstrated the least amount of rocking and pressure fluctuations encountered at the surgical sites when lying supine. These findings addressed clinicians’ concerns, that using the higher resistance and lower rotational speeds would minimize potential sheering at the wound site. Further, because the exercise was performed while lying supine, the surgeon and certified wound nurse insisted that for participants with an ischial flap initiating exercise would be post-operative day 7 or later; and for those who had sacral or coccyx flap surgery the onset of arm bike use would be post-operative day 14 or later. They also reserved the right to postpone or cancel exercise with the M-PACE if the surgical wound was not healing as expected. To address concerns that using the arm ergometer while lying flat in bed may harm the surgical wound, the study team therapists checked the surgical wound pre- and post-exercise, every session. Because no adverse changes were noted by the therapists, the study team, including the surgeon and wound nurse, were reassured the pre-screening process as described previously, was appropriate. Further, several team members wonder if the exercise may actually improve length of stay. This question will need to be answered by a larger randomized trial.

The study team was careful to pre-screen and exclude patients who our clinical team thought might not be a good fit for using the supine arm cycle ergometer post-operatively. The percentage of patients determined by the clinical staff to be appropriate to use the M-PACE (67%) demonstrates that the clinical team was careful to screen for who should or should not use the M-PACE for maintaining their conditioning while on complete post-operative bedrest. As mentioned earlier, factors such as poor skin integrity, unmitigable pain in arm(s) or shoulder(s), poor arm function, inappropriate behavior, or the patient was not interested in participating were some of the barriers to enrollment as determined by the clinical staff. Once enrolled, the clinical team always had the option to withdraw the participant from the study if they thought there might be potential for harm.

The participant response to using the M-PACE was quite positive. Although interpretation of the feedback needs to be carefully considered. There may have been more interaction with the therapists using the arm bike versus a standard of care using the TheraBands^®^ or weights.

The 6MAT data tell us that there may be some beneficial conditioning effects of performing arm cycling while on bedrest. Though there are not enough data to determine dosage needed to make a difference, 14 of 15 participants either maintained or improved their conditioning per reported RPE during their bedrest. This alone, is interesting because we have known for a long time the detrimental effects of prolonged bed rest, including deconditioning.^1,2^ Yet, with exercise in this study we were able to show that stopping deconditioning and even reconditioning is possible in this population during the post-flap recovery period. In addition to reduced RPE, many subjects produced more rotations of the arm crank during the 6MAT (*P* = 0.02), suggesting they did more work with less perceived exertion. Ideally, the number of rotations would be kept constant between pre- and post-conditions. Both changes, the RPE and the number of rotations, suggest reductions in deconditioning.

The study team acknowledges there are limitations to the methods and interpretations of the data stemming from a lack of equipoise among the study team. From the beginning there was a belief that exercise would be beneficial. This became apparent when planning a randomized trial. The team was ethically unable to say they would be willing to randomize a patient to not getting access to the arm bike post-operatively if they had no clinical contraindications for using it. Thus, we decided to study whether deconditioning could be stopped or reversed rather than using a stronger randomized controlled trial (RCT). Other groups with equipoise may choose to run a stronger RCT in the future.

Another limitation to the interpretation is that the study was conducted by the MADE personnel, the same group that invented the device. The study was initiated by two of the device’s inventors (AH and GD) but was later handed off to the primary author (CMO) who is not an inventor. All 6MAT testing was led by non-inventors of the machine and exercise testing was conducted by clinical therapists in SCI who were also not inventors. Future testing at other facilities not involved in the development may provide additional information regarding the M-PACE.

## Conclusion

In this pilot study, the 6MAT RPE pre- to post data demonstrate that deconditioning while on extended bedrest was offset when the M-PACE was used by patients recovering from flap surgery. Further, many of the study participants who accessed the M-PACE found using the arm bike helped offset the tedium of laying supine during flap surgery recovery. The differences in the 6MAT pre- to post-measures indicate the supine arm cycle ergometer should be further studied for offsetting the normal deconditioning that occurs with extended bedrest. Besides using the outcome measures from this pilot study, for a future randomized trial other suggested outcome measures to include: time to return to normal ADLs; length of stay; one-year flap failure rate.
